# Kawasaki disease associated with Mycoplasma pneumoniae

**DOI:** 10.1186/s13052-016-0292-1

**Published:** 2016-09-08

**Authors:** Yunjia Tang, Wenhua Yan, Ling Sun, Jie Huang, Weiguo Qian, Miao Hou, Haitao Lv

**Affiliations:** Department of Cardiology, Children’s Hospital of Soochow University, Suzhou, China

**Keywords:** Mycoplasma pneumoniae, Kawasaki disease, Children

## Abstract

**Background:**

Kawasaki disease (KD) is an illness of unknown etiology that mostly occurs in children under 5 years of age and is the leading cause of acquired heart disease all over the world. *Mycoplasma pneumoniae* (MP) was one of the likely causative agents of KD. However, the etiologic effect of MP in KD has not been fully recognized.

**Methods:**

We prospectively analyzed the clinical records of 450 patients with KD hospitalized in Children’s Hospital of Soochow University from 2012 to 2014. Using medical records, we retrospectively identified patients with low respiratory tract infection (non-KD group).

**Results:**

Of the 450 KD patients, MP was positive in 62 (13.8 %). The median age of the MP + KD+ group was significantly older than the MP-KD+ group (25 vs 14.5 months, *P* < 0.01). MP + KD+ group had higher levels of ESR, N% and CRP than the MP-KD+ group. MP + KD+ group were more frequent in respiratory disorders than MP-KD+ group with a *P* < 0.05. No statistical difference of non-responders or coronary artery lesion was found between the groups.

**Conclusions:**

MP infections are found in an important proportion of the KD patients (13.8 % in our series). MP infection tended to occur in older populations and with a higher rate of respiratory tract involvement in patients with KD. No statistical difference of non-responders or coronary artery lesion was found between the MP+ and MP- KD patients.

## Background

Kawasaki disease (KD) is a self-limited vasculitis characterized by fever ≥5 days, conjunctive injection, changes in extremities, oral mucosal changes, rash, and cervical lymphadenopathy. The main complication of the febrile disease is coronary artery lesion (CAL), which makes the disease the major cause of acquired heart disease in developed countries [1]. The etiology of KD remains unknown though nearly fifty years have passed since the first report of the disease in 1967 [2]. Infection is considered to be one of the predisposing factors [3–5]. But despite numbers of investigations and multiple candidates, no unique infectious agent has been identified as the sole etiologic agent responsible for KD so far.

There are a few KD cases reported to be associated with the etiologic agent of *Mycoplasma pneumoniae* (MP) [6–8], but no systemic study on MP infected (MP+) KD patients was carried out so far. Thus the etiologic effect of MP in KD is of particular interest.

The present study was designed to determine the clinical and epidemiological characteristics and CAL in MP+ KD patients and to reveal the differences between the MP+ and non-MP infected (MP-) KD patients.

## Methods

### Study patients

We conducted a prospective study in Children’s Hospital of Soochow University during January 1^st^, 2012 and December 31^th^, 2014. A standardized protocol was used to enroll a target number of consecutive patients from the inpatient ward of cardiology. Patients with a diagnosis of KD were eligible for inclusion. On admission, after obtaining informed consent from the parents, clinical-epidemiological information was collected by pediatricians. Using medical records, we retrospectively identified patients with low respiratory tract infection (LRTI) (non-KD group) from the ward of pulmonology. All experiments were performed following the relevant guidelines and regulations of Soochow University. The study was approved by the Medical Ethics Committee of Soochow University.

### Data collection

Data such as age, sex, season onset, fever duration before diagnosis, length of stay in hospital, KD-related clinical manifestations and other systemic involvements, white blood cell (WBC) count, platelet (PLT), neutrophils proportion (N%), c-reaction protein (CRP), erythrocyte sedimentation rate (ESR), MP antibody, MP-DNA by PCR and echocardiographic findings within 1 month of onset were collected and further analyzed. WBC, PLT, N%, CRP and ESR were tested within 24 h after admission.

### MP-DNA detection and evaluation

Nasopharyngeal secretions were collected from each study participant within 24 h after admission by a lab technician as previously described [9]. Briefly, an aseptic plastic sputum catheter was inserted into the nostril to a depth of about 7–8 cm until reaching the pharynx. Approximately 2 ml of nasopharyngeal secretions was collected by applying negative pressure. The sample was mixed with 4–8 ml PBS, and centrifuged for 10 min at 300–500 rpm. The supernatant was discarded and the pellet was mixed with 4–8 ml PBS and centrifuged for an additional 10 min. The pellet was stored at 280uC until testing began.

DNA lysate (Shanghai Shenyou biotechnology company, Shanghai, China) was added to the sputum pellet following washing with PBS. The sample was heated to at 95 °C for 10 min, centrifuged for 5 min at 12 000 rpm, and then the supernatant was collected. After extracting the DNA from the sputum specimen, MP DNA was detected by fluorescent real-time PCR (BIO-RAD iCycler, USA). The cyclic temperature settings were 93 °C, 2 min; 93 °C, 45 s; 55 °C, 60 s → 10 cycles; 93 °C 30 s → 55 °C, 45 s → 30 cycles. The fluorescence collection point was set at the 55 °C, 45 s. Ct value was used to quantify the fluorescence quantitative PCR results. The following primers were used: The probe binding sequence was located between the upstream and downstream primer. The fluorescent reporter dye at the 59 end of probe was 6-carboxyfluorescein, and the quencher at the 39 end of the probe was 6-carboxytetramethylrhodamine. The primers and probe were purchased from Guangzhou Daan Gene Ltd. (Guangzhou, China). An MP-negative sample was defined as having an amplification curve that was not S-shaped or a Ct value = 30. Both results indicated that the MP DNA content was below the detection limit. A positive MP sample was defined as having an amplification curve that was S-shaped and a Ct value <30.

### Serological analysis for MP

Paired serum samples were collected on admission and at least 1 week later. IgM antibodies were measured using the Serion Elisa Mycoplasma pneumoniae IgM kit (Institut Virion/serion GmbH, Würzburg, Germany) with the test cut-off 0.5 × mean optical density value of the kit control serum, as indicated in the insert. The assay was considered positive if IgM ≥1.1U/ml according to manufacturer instructions.

### Diagnosis of MP infection

Diagnosis of MP infection was based on serology and PCR findings. Both the presence of IgM antibodies and positive PCR results were used as sufficient criteria for current MP infection.

### Definition of KD

KD was defined by the presence of ≥5 days of fever and ≥4 of the 5 principal clinical features for KD according to American Heart Association [1]. These clinical features included (1) bilateral non-exudative conjunctival injection; (2) oral mucosal changes, such as erythema of the lips or strawberry tongue; (3) changes in extremities, such as edema, erythema and desquamation; (4) polymorphous rash and (5) cervical lymphadenopathy of ≥1.5 cm. Patients with only two or three principal clinical features of KD, in addition to fever, are considered to have incomplete KD when CAL was confirmed. CAL (in echocardiography) was defined as an internal lumen diameter ≥3 mm in children <5 years of age or ≥4 mm in children >5 years of age. Coronary artery aneurysm (CAA) was defined as a segmental internal diameter of any segment ≥1.5 times greater than that of an adjacent segment. Giant coronary aneurysm was referred to a segmental internal diameter ≥8 mm [10]. Non-responders were referred to persistent or recrudescent fever ≥36 h after the initial IVIG infusion.

### Statistical analysis

We used n (%) for categorical variables and median (quartiles) for continuous variables with non-normal distribution or mean and standard deviation (SD) for those with normal distribution. We assessed differences in categorical variables with the *χ*^2^ test. We calculated 95 % confidence interval (95 % CI) for differences in medians with an exact test. Logistic regression analysis was performed to identify different clinical characteristics and laboratory parameters associated with MP infection. SPSS (version 22.0) software was used for all statistical analysis.

## Results

### Clinical features and laboratory parameters of the patient groups

A total of 483 patients were diagnosed with KD. Thirty-three patients were excluded because of incomplete data. The remaining 450 patients were enrolled in this study. The age of onset ranged from 2 to 129 months with a median of 17 months old. The male to female ratio was 1.86:1. KD criteria of diagnosis included fever in 100 % of the patients, rash in 76.2 %, conjunctival injection in 84.2 %, changes in extremities in 77.6 %, cervical lymphadenopathy in 61.1 % and mucosal changes in 88 %.

Of the 450 KD patients, MP was positive in 62 (13.8 %) patients. The MP + KD+ group consisted 62 cases while the MP-KD+ group consisted 388 cases. The clinical features and laboratory parameters of the two groups are shown in Table 1. The median age of the MP + KD+ group was significantly older than the MP-KD+ group (25 vs 14.5 months, *P* < 0.01). MP + KD+ group had higher levels of ESR, N% and CRP than the MP-KD+ group with significant differences after logistic regression analysis.

**Table 1:** Clinical features and laboratory parameters of children with Kawasaki disease with or without Mycoplasma pneumoniae infection

	MP + KD+ Group	MP-KD+ Group	*P* value
No. of patients (%)	62 (13.7)	388 (86.3)	-
Median age (quartiles), m	25 (17, 48)	14.5 (8, 29)	<0.01*
Male to female ratio	1.82	1.91	0.62
Median fever duration before the diagnosis of KD (quartiles), days	7 (5, 8)	6 (5, 7)	0.14*
Rash, n (%)	49 (79.0)	294 (75.8)	0.57
Conjunctival injection, n (%)	50 (80.6)	323 (83.2)	0.61
Changes in extremities, n (%)	51 (82.3)	298 (76.8)	0.34
Cervical lymphadenopathy, n (%)	43 (69.4)	233 (60.1)	0.16
Mucosal changes, n (%)	55 (88.7)	340 (87.6)	0.81
ESR, mm/h	39.9 ± 25.4	35.4 ± 24.0	0.03*
WBC, ×10^9^/L	14.6 ± 6.4	14.1 ± 5.0	0.45
PLT, ×10^9^/L	369.8 ± 130.1	381.5 ± 125.4	0.30
N%	68.6 ± 13.8	64.0 ± 15.9	<0.01*
CRP, mg/L	70.6 ± 15.6	64.7 ± 15.1	<0.01*

*Median age, median fever duration before the diagnosis of KD,ESR, N% and CRP were tested by logistic regression analysis to exclude the interaction betweenparameters

A total of 6354 patients were included in the non-KD group. MP was positive in 1302 (20.5 %) patients. The median age of the 1302 children were 35 months. The ratio of male to female was 1.46:1. The median age of patients with MP infection in KD group were significant younger than that in non-KD group (25 vs 35 months, *P* < 0.001). The clinical features and laboratory parameters between the two groups are shown in Table 2.

**Table 2:** Clinical features and laboratory parameters of MP infection children with KD and non-KD disease

	KD (*n* = 62)	Non-KD (*n* = 1302)	*P*
Median age (quartiles), m	25 (17,48)	35 (10,56)	<0.01
Male to female ratio	1.82	1.46	0.44
Median length of stay in hospital (quartiles), days	10 (8, 12)	7 (5–10)	<0.01
WBC, ×10^9^/L	14.6 ± 6.4	7.7 ± 4.3	<0.01
PLT, ×10^9^/L	369.8 ± 130.0	274.0 ± 101.0	<0.01
N%	68.6 ± 13.8	51.1 ± 12.3	<0.01
CRP, mg/L	70.6 ± 15.6	22.6 ± 20.8	<0.01

### Epidemiology of MP infection in KD and non-KD patients

**Fig. 1:**
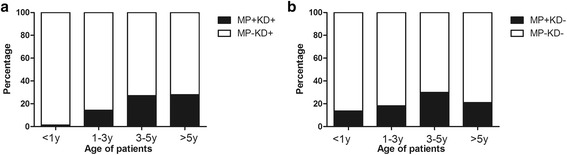
Age distribution of MP infection in KD (**a**) and non-KD (**b**) patients

The MP infection rate in KD patients increased with their age, with a statistical significance for age distribution (*P* < 0.001). Fewer children younger than 1 year old had MP infection (1.3 %) than those older than 5 years old (28 %) (*P* < 0.001) (Fig. 1a), indicating that the older children with KD were more prone to MP infection. The age distribution of MP infection in KD group was similar to that in non-KD group (Fig. 1b).

**Fig. 2:**
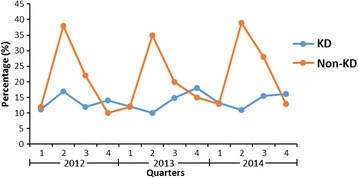
Seasonal distribution of MP infection in KD and non-KD patients

The seasons in the Suzhou area of China were defined as spring (March–May), summer (June–August), autumn (September–November), and winter (December–February). During the 3-year period, seasonal discrepancy of MP infection was observed in non-KD group. MP was detected throughout the year with an epidemic peak observed each year in summer season. In KD group, the highest proportion of KD onset occurred during March through July (53 %), with a peak in May. However, seasonal discrepancy of MP infection was not observed in KD patients (Fig. 2).

### Response to treatment in KD patients

A total dose of 2 g/kg of intravenous immunoglobulin (IVIG) was administered in patients in 432 patients, mainly during the 5^th^ and 9^th^ day of the disease onset (98.5 %). Among the patients who did not received IVIG (initial missed diagnosis and low level of IgA), 7 belonged to the MP + KD+ group and 11 belonged to the MP-KD+ group. An additional high dose of aspirin of 30–50 mg/kg was used during the febrile period and thereafter a low dose of 3–5 mg/kg was administrated until afebrile for 3 days. Non-responders were administered a second dose of 2 g/kg of IVIG. Intravenous pulse methylprednisolone was administered in 15 (71.4 %) of the non-responders with a regimen of 2 mg/kg/d for 3 to 5 days. Overall, 411 (95.2 %) patients responded to the initial IVIG treatment and all non-responders (21 patients, 4.8 %) responded to the second IVIG treatment with or without methylprednisolone. Of the 21 non-responders, 3 (4.8 %) were MP+ and 18 (4.6 %) were MP- patients. No statistical difference of non-responders was found between the groups (OR: 1.314, 95 % CI: 0.553–3.236). Intravenous pulse or oral azithromycin of 10 mg/kg/d for consecutive 3–5 days was administered in 20 of the MP+ patients with obvious respiratory tract signs and symptoms.

### Length of hospitalization and coronary artery lesion in KD patients

We compared length of hospitalization of the MP+ and MP- KD patients. 92.1 % of the patients discharged within 15 days of hospitalization. No significant difference was found in the length of the hospitalization between the MP+ and MP- KD patients. Besides, no significant difference was found in the length of hospitalization in the patients with azithromycin and without azithromycin treatment (10 [9, 12], 10 [8, 12], respectively, *P* > 0.05).

CAL was found in 108 (24 %) of the patients, most of whose abnormalities was dilation only (88.0 %). No difference was found of CAL between the two groups. These findings are shown in Table 3.

**Table 3:** Coronary artery lesions and length of hospitalization in Kawasaki disease patients with or without Mycoplasma pneumoniae infection

	Total (*n* = 450)	MP + KD+ group (*n* = 62)	MP-KD+ group (*n* = 388)	*P* value
CAL total, n (%)	108 (24.0)	15 (24.2)	93 (24.0)	0.97
Dilation only, n (%)	95 (21.1)	12 (19.4)	83 (21.4)	0.13
Aneurysm (≥4 mm), n (%)	18 (4.0)	3 (4.8)	15 (3.9)	0.72
Giant aneurysm (≥8 mm), n (%)	1 of 18	0 of 3	1 of 15	1.00
Median length of stay in hospital (quartiles), days	10 (8, 12)	10 (8, 12)	10 (8, 12)	0.65

### Other systemic involvement in KD patients

Many patients in KD presented with other systemic involvements besides CAL. These results are seen in Table 4. Respiratory and gastrointestinal disorders were most commonly seen in both groups (48.21 % and 37.97 %, respectively). Respiratory disorders mainly included rhinorrhea, sore throat, cough, sputum and wheeze. Gastrointestinal disorders mainly included anorexia, vomiting, diarrhea and abdominal pain. Urinary system involvement included urethritis and meatitis. MP + KD+ group were more frequent in respiratory disorders than MP-KD+ group with a *P* < 0.05. On the other hand, there was no difference in the rate of gastrointestinal tract, urinary system involvement, aseptic meningitis and hepatic dysfunction. Besides, other complications like paroditis, arthritis, myocarditis and arrhythmia were seen in few cases.

**Table 4:** Other systemic involvement with or without Mycoplasma pneumoniae infection

	Total (*n* = 450)	MP + KD+ group (*n* = 62)	MP-KD+ group (*n* = 388)	*P* value
Respiratory tract, n (%)	181 (40.2)	32 (51.6)	149 (38.4)	0.04
Gastrointestinal tract, n (%)	77 (17.1)	13 (20.1)	64 (16.5)	0.38
Urinary system, n (%)	49 (10.9)	7 (11.3)	42 (10.8)	0.91
Aseptic meningitis, n (%)	17 (3.8)	3 (4.8)	14 (3.6)	0.72
Hepatic dysfunction, n (%)	83 (18.4)	9 (14.5)	74 (19.1)	0.39

Aseptic meningitis was diagnosed by lumbar puncture

Hepatic dysfunction was diagnosed when ALT is ≥ 80U/L when other hepatic diseases were excluded

## Discussion

The etiology of KD remains unclear up to now despite numbers of studies. Many case reports have found that infectious agents might play an important role [8, 11–15]. The mechanism of infectious agents associated with KD has not been fully understood. Abe et al. [16] suggested that the process of superantigens activating T cell might be important in the pathophysiology of KD. Another species of the Mycoplasma genome, *Mycoplasma arthritidis*, has been shown to produce a superantigen suggesting the possibility that other Mycoplasma species, like MP, may do likewise [17]. MP was one of the most reported organisms to cause KD [7, 8], but the clinical characteristics of MP infected KD have not been thoroughly examined. As far as we know, this is by far the largest study to determine the role of MP infection in KD.

In our study, MP + KD+ patients were much older than MP-KD+ patients which was in accordance with Lee MNet al. [8]. In their research, they found that the MP group was significantly older than the non-MP group in KD patients (5.5 ± 3.5 vs 2.8 ± 2.2 years). In our study, we also compared the median age of patients with MP infection in KD and non-KD groups. Patients with MP infection in KD were significantly younger than those in non-KD group (25 vs 35 months, *P* < 0.001), although the age distribution of patients with MP is similar between KD and non-KD group.

Seasonal peaks of MP infection in LRTI has been reported in our previous studies. Outbreaks began in summer months and peaked during August and September in Suzhou [9, 18]. The seasonal distribution in our non-KD group is in line with our previous studies. However, interestingly, no seasonal peaks of MP infection in KD was observed in our study. This phenomenon imply that outbreaks of MP infection in LRTI may not increase the incidence of MP associated KD.

There was no significant difference in the incidence of CAL in our study. Lee et al. and his colleagues also found no difference of both left and right coronary artery in MP+ and MP- KD patients by analyzing 12 KD patients and 42 controls [8]. Though MP as an important causative agent that mainly resides in the respiratory tract, it may disseminate systemically to the peripheral blood mononuclear cells and localize in arteries where it may infect endothelial cells, vascular smooth muscle cells and monocytes/macrophages, leading to vascular changes. In earlier years Momiyama Y et al. proposed an elevated level of MP antiboby was associated with coronary artery disease [19], suggesting a closed relationship between MP and vascular changes. Unfortunately, there was no difference of CAL or CAA between MP+ and MP- KD patients in our study. But it need to be verified by larger study samples.

KD itself can lead to respiratory, gastrointestinal, urinary, hepatic and nervous systems disorder [1]. In our study, we found that respiratory involvement was more common in MP + KD+ group compared with MP-KD+ group. Respiratory involvement in KD might be a consequence of vessel inflammation with increased vascular permeability and perivascular edematous changes [20]. It is confirmed that MP infections involve both the upper and lower respiratory tract, which could partly explain why respiratory tract involvement was observed frequently in the MP + KD+ patients. Besides, MP is also reported to be responsible for non-respiratory tract manifestations, including neurological, hepatic and cardiac diseases [21–23]. However, there was no statistical difference in terms of non-respiratory manifestations between MP+ and MP- KD patients. This might indicate that MP plays a minor role in these systems in KD patients.

In MP infection, it is difficult to set up criteria for the “gold standard” to detect acute infections. Cultural isolation is 100 % specific but too slow to be of timely diagnostic value. There is no universally agreed upon gold standard serological assay for detection of antibodies to MP. Paired serology (≥4-fold rise in IgG titer by complement fixation tests) remains the mainstay for diagnosing MP [24]. However, our patients were treated with high-dose IVIG that contain substantial amounts of MP antibody. Such passive immunization would greatly increase MP-IgG. Based on this, in our study we detect MP-IgM, instead of IgG, to diagnose MP infection. On the other hand, specific IgM antibody could persist for up to a year after infection in some patients, but the pathogen is detected less frequently during the later stages of the disease. In our study, both the presence of IgM antibodies and positive PCR results were used as sufficient criteria of current MP infection. So we believed that our definition of MP infection has great accuracy in determining MP infection in KD patients.

There are some limitations in the present study. First, the size of the study population was relatively small. Therefore, more studies are needed in order to firmly establish the relationship between simple MP infection and KD. In addition, overdiagnosis and underdiagnosis may exist for the lack of golden diagnostic criteria for KD and the similarity between MP infection and KD.

## Conclusion

We demonstrated 13.8 % patients had MP infection at the time of KD diagnosis. KD patients with MP infection tended to occur in older populations and with a higher rate of respiratory tract involvement. No statistical difference of non-responders or coronary artery lesion was found between the MP+ and MP- KD patients.

## Abbreviations

KD: Kawasaki disease
MP: *Mycoplasma pneumonia*
CAL: Coronary artery lesion
WBC: White blood cell
PLT: Platelet
N%: neutrophils proportion
CRP: c-reaction protein
ESR: Erythrocyte sedimentation rate
IVIG: Intravenous immunoglobulin
LRTI: Low respiratory tract infections
